# Characterization of virus species associated with sweetpotato virus diseases in Burkina Faso

**DOI:** 10.1111/ppa.13190

**Published:** 2020-04-30

**Authors:** Ezechiel B. Tibiri, Justin S. Pita, Fidèle Tiendrébéogo, Martine Bangratz, James B. Néya, Christophe Brugidou, Koussao Somé, Nicolas Barro

**Affiliations:** ^1^ Laboratoire de Virologie et de Biotechnologies Végétales Institut de l’Environnement et de Recherches Agricoles (INERA) Ouagadougou Burkina Faso; ^2^ Laboratoire de Génétique et de Biotechnologies Végétales Institut de l’Environnement et de Recherches Agricoles (INERA) Ouagadougou Burkina Faso; ^3^ Laboratoire Mixte International Patho‐Bios IRD‐INERA Ouagadougou Burkina Faso; ^4^ Laboratoire d’Epidémiologie et de Surveillance des bactéries et virus Transmissibles par les Aliments et l’eau LabESTA/UFR/SVT Université Joseph Ki‐Zerbo Ouagadougou Burkina Faso; ^5^ Central and West African Virus Epidemiology (WAVE), Pôle Scientifique et d’innovation de Bingerville Université Félix Houphouët‐Boigny (UFHB) Bingerville Côte d’Ivoire; ^6^ Interactions Plants Microorganismes et Environnement (IPME) IRD, Cirad Université Montpellier Montpellier Cedex France

**Keywords:** diagnostic, grafting, *Ipomoea batatas*, *Ipomoea setosa*, SPVD, sweetpotato

## Abstract

Sweetpotato (*Ipomoea batatas*) production in sub‐Saharan Africa is severely affected by viral diseases caused by several interacting viruses, including sweet potato feathery mottle virus (SPFMV), sweet potato chlorotic stunt virus (SPCSV), and sweet potato leaf curl virus (SPLCV). However, the aetiology of viral symptoms on sweetpotato is rarely established in most countries in Africa. Here, we aimed to investigate and characterize the incidence of sweetpotato viruses in Burkina Faso. We performed a countrywide survey in 18 districts of Burkina Faso and collected 600 plants, with and without symptoms, from 80 fields. Viral strains were identified using nitrocellulose membrane‐ELISA, PCR, and reverse transcription‐PCR. Three scions from each of 50 selected plants with symptoms were grafted to healthy *Ipomoea setosa* and then serological and molecular tests were performed on the 150 recorded samples. Three viruses were detected: 24% of samples were positive for SPFMV, 18% for SPLCV, and 2% for SPCSV. Across all diagnostic tests, 40% of all plant samples were virus‐negative. Coinfections were found in 16% of samples. Partial sequences were obtained, including 13 that matched SPFMV, one that matched SPLCV, and one that matched SPCSV. All identified SPFMV isolates belonged to either phylogroup B or A‐II. The SPCSV‐positive isolates had 98% gene sequence homology with SPCSV‐West Africa for the coat protein. Begomovirus‐positive isolates clustered with SPLCV‐United States. This first study of sweetpotato viral diseases in Burkina Faso indicates widespread occurrence and suggests a need for further epidemiological investigations, breeding programmes focused on virus‐resistant varieties, and improved farming practices to control disease spread.

## Graphical abstract

We investigated the viral disease threatening sweetpotato production in Burkina Faso. SPFMV, SPLCV and SPCSV were found; these viruses can occur together with severe symptoms.

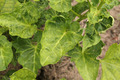

## 1 INTRODUCTION

In sub‐Saharan Africa, sweetpotato (*Ipomoea batatas*) is the third most important root and tuber crop after cassava (*Manihot esculenta*) and yam (*Dioscorea* spp.). This highly productive crop performs well in poor soils and is relatively drought‐insensitive (Tairo *et al.*, 2005). However, sweetpotato productivity is constrained by both biotic and abiotic factors; of these, viral diseases and weevil damage are by far the most important (Valverde *et al.*, 2007). More than 30 viruses have been reported to infect sweetpotato, and several of these are recently described DNA viruses belonging to the families *Geminiviridae* and *Caulimoviridae* (Clark *et al.*, 2012; Cuellar *et al.*, 2015).

In Burkina Faso, sweetpotato is the most important root crop and ranks third, after cereals and legumes, among all crops (Dabiré and Belem, 2001). Sweetpotato production in Burkina Faso increased from 13,618 t in 1998 to 167,550 t in 2013 (FAOSTAT, 2013). In western Burkina Faso, sweetpotato is grown as a cash crop. However, because sweetpotato is propagated vegetatively, it is prone to the accumulation of viruses and other pathogens (Souto *et al.*, 2003; Cuellar *et al.*, 2015). Farmers can lose up to 100% of the crop yield; losses differ depending on the cultivar, the infecting virus, the stage of infection, and whether the crop is infected with one or more viruses (Valverde *et al.*, 2007; Rey *et al.*, 2012; Loebenstein, 2015). Given the susceptibility of sweetpotato to virus infection, coinfections are common and cause a generalized disease known as sweet potato virus disease (SPVD; Valverde *et al.*, 2007).

Sweet potato feathery mottle virus (SPFMV; family *Potyviridae*, genus *Potyvirus*) is one of the most damaging viruses infecting sweetpotato worldwide (Moyer *et al.*, 1980; Untiveros *et al.*, 2008; Loebenstein, 2015). The virus is transmitted by aphids, including *Aphis gossypii*, *A*. *craccivora*, *Myzus persicae*, and *Lipaphis erysimi*, in a nonpersistent manner (Ward and Shukla, 1991; Loebenstein, 2015). Like many sweetpotato viruses, SPFMV can also be mechanically transmitted through grafting to *Ipomoea* spp., such as *I*. *batatas*, *I*. *setosa*, *I*. *nil*, *I*. *incarnata*, and *I*. *purpurea*, as well as to *Nicotiana benthamiana* and *Chenopodium amaranticolor* (Clark *et al.*, 2012). Transmission of SPFMV by seed, pollen, or contact between plants has not been proven (Clark *et al.*, 2012; Loebenstein, 2015). SPFMV can be diagnosed by grafting infected scions onto *I*. *setosa*, on which it causes vein‐clearing and vein‐banding (Rännäli *et al.*, 2009; Loebenstein, 2015).

The SPFMV genome consists of a single positive‐sense RNA strand with a poly(A) tail at its 3′ terminus and a genome‐linked protein (VPg) at its 5′ terminus (Wylie *et al.*, 2017). The genome contains a large open reading frame (ORF) that encodes a polyprotein and the PIPO ORF (Wylie *et al.*, 2017). Previous studies on the diversity of the coat protein (CP) genomic region revealed four phylogenetic lineages: East African (EA) from Tanzania, Uganda, and Kenya; russet crack (RC) from Australia, Africa, Asia, and North America; ordinary (O) from Japan, China, Korea, Niger, Nigeria, and Argentina; and sweet potato virus C (as renamed by Adams and Carstens, 2012) from the USA, China, Australia, Africa, East Africa, and Argentina (Untiveros *et al.*, 2008; Adams and Carstens, 2012). With increased understanding of SPFMV and the biosafety implication of its nomenclature, a neutral classification system has been proposed that uses Latinized numerals to take account of biological and geographical considerations (Jones and Kehoe, 2016). Consequently, SPFMV EA became A‐I, O became A‐II, and RC became B (Maina *et al.*, 2018). SPFMV has been characterized in East Africa (Kenya, Tanzania, and Uganda) and West Africa (Nigeria and Niger), where it is a known threat to sweetpotato production (Karyeija *et al.*, 2001; Untiveros *et al.*, 2008).

The begomoviruses known to infect sweetpotato (“sweepoviruses”) are all monopartite and phylogenetically distinct from both the Old and New World begomovirus species (Fauquet and Stanley, 2005). In Africa, sweepoviruses have been reported only in Kenya, Uganda, South Africa, Tanzania and, more recently, in Sudan (Miano *et al.*, 2006; Wasswa *et al.*, 2011; Esterhuizen *et al.*, 2012; Mbanzibwa *et al.*, 2014; Mohammed *et al.*, 2017). Sweepoviruses are transmitted by the whitefly *Bemisia tabaci* (Trenado *et al.*, 2011).

Sweet potato chlorotic stunt virus (SPCSV) is a bipartite member of the family *Closteroviridae*, genus *Crinivirus*. Like other criniviruses, it is phloem‐limited (Cohen *et al.*, 1992; van Regenmortel *et al.*, 2000; Loebenstein, 2015). SPCSV has a worldwide distribution (Karyeija *et al.*, 1998) and is present in the main sweetpotato production areas in Africa, and usually coinfects its host plant with other viruses in the field (Mukasa *et al.*, 2003; Tairo *et al.*, 2004). Coinfection makes separation and purification of SPCSV difficult, thus determination of the SPCSV genomic sequence has been greatly constrained (Qin *et al.*, 2014). To date, only two SPCSV strains have been identified: SPCSV‐East African (SPCSV‐EA) and SPCSV‐West African (SPCSV‐WA). The SPCSV‐EA strain is more widespread and more studied than SPCSV‐WA. Studies have shown that most sweetpotato‐infecting viruses can cause severe synergistic disease complexes when they coinfect with SPCSV, leading to increased yield losses. These synergistic disease complexes may include RNA viruses of genera *Potyvirus*, *Ipomovirus*, *Carlavirus*, and *Cucumovirus*, as well as DNA viruses of genera *Begomovirus*, *Cavemovirus*, and *Solendovirus*, that are symptomless when they infect singly (Karyeija *et al.*, 2000; Mukasa *et al.*, 2006; Untiveros *et al.*, 2007; Cuellar *et al.*, 2011; 2015). Thus, SPCSV can increase the susceptibility of sweetpotato to a wide range of viruses (Cuellar *et al.*, 2009; 2015).

In coinfection with SPCSV, SPFMV can cause SPVD, which is responsible for yield losses of 70%–100% (Milgram *et al.*, 1996; Gutiérrez *et al.*, 2003; Ateka *et al.*, 2004; Mukasa *et al.*, 2006; Untiveros *et al.*, 2008; Loebenstein, 2015). Additionally, molecular studies showed that coinfection with SPCSV enhances SPFMV RNA viral titres at least 600‐fold, whereas SPCSV titres remain equal or less compared with single infection (Karyeija *et al.*, 2000; Kokkinos and Clark, 2006; Mukasa *et al.*, 2006).

As Burkina Faso is now actively including orange‐fleshed sweetpotato in its food security strategy, there is increased focus on major threats to sweetpotato production, particularly from viruses. Symptoms resembling those induced by SPVD have been observed in sweetpotato fields in the main production areas in Burkina Faso, but the viruses associated with SPVD have never been confirmed. A study in 2010 by the Pan‐African Sweet Potato Virome project (http://bioinfo.bti.cornell.edu/virome) aimed to understand the diversity, distribution, and evolution of sweetpotato viruses across Africa. However, only a few samples from one site in Burkina Faso were available to that project. Nevertheless, this small sample identified the presence of sweet potato leaf curl virus (SPLCV), SPCSV, and SPFMV.

The aim of our countrywide study in Burkina Faso was to assess the presence of viruses in sweetpotato‐growing areas and to establish the aetiology of the symptoms observed in the sweetpotato fields surveyed. The knowledge generated will contribute to the current body of knowledge of sweetpotato viruses and will inform decisions concerning production of this crop in Burkina Faso.

## 2 MATERIALS AND METHODS

### 2.1 Plant material collection

**Figure 1:**
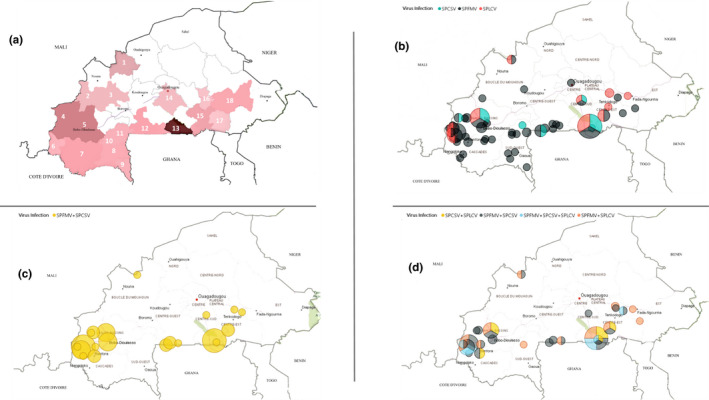
Sweetpotato survey 2015–2017: (a) Map of Burkina Faso showing the locations of samples in this study. Areas sampled for sweetpotato leaves and cuttings are listed in Table 1. (b) Locations with samples positive for SPCSV, SPFMV, and SPLCV by molecular diagnostic methods. The smallest, single‐colour circles represent one specific virus identified at one farm; medium single‐colour circles indicate one specific virus identified at more than one farm; largest segmented circles indicate the detection of more than one virus at more than one farm. Viruses detected in each location are shown in Table 1. (c) Locations with samples positive for mixed infection of SPFMV + SPCSV by molecular diagnostic methods. The smallest circles represent one farm with SPFMV + SPCSV infections; medium circles indicate >1 farm with SPFMV + SPCSV infections. Locations with samples positive for mixed infections of SPFMV + SPCSV are shown in Table 1. (d) Locations with samples positive for mixed infection of SPCSV, SPFMV, and SPLCV by molecular diagnostic methods. Within the areas sampled, mixed infections were recorded. The smallest, single‐colour circles represent one specific virus combination identified at one farm; the smallest, segmented circles represent detection of more than one virus combination at one farm; larger single‐colour circles indicate one specific virus combination found at more than one farm; larger segmented circles indicate the detection of more than one virus combination at more than one farm. Mixed infections detected in each location are shown in Table 1 [Colour figure can be viewed at wileyonlinelibrary.com]

From March 2015 to October 2017, 600 young (top) leaves and stem cuttings exhibiting typical symptoms as well as symptomless ones were collected from 80 farms in 18 provinces in Burkina Faso (Figure 1a, Table 1). The leaf samples were immediately put into paper envelopes, dried at 37 °C, and stored at the Environmental and Agricultural Research Institute laboratory located at Kamboinsé Research Station. Cuttings (10–15 cm length) were grown in plastic pots containing an autoclaved mixture of soil, sand, and peat moss (equal volumes) and maintained in an insect‐proof greenhouse.

**Table 1:** Results of nitrocellulose membrane‐ELISA, reverse transcription‐PCR, and PCR on sweetpotato showing SPFMV, SPLCV, and SPCSV in single infection and SPFMV + SPCSV (sweet potato virus disease, SPVD), SPFMV + SPLCV, SPCSV + SPLCV, and SPFMV + SPCSV + SPLCV in mixed infection by locality in Burkina Faso

Province	Sweetpotato plants infected with							
	SPFMV	SPLCV	SPCSV	SPFMV + SPCSV (SPVD)	SPFMV + SPLCV	SPCSV + SPLCV	SPFMV + SPCSV + SPLCV	Plants uninfected
Sourou	4	8	0	2	1	0	0	2
Banwa	2	0	0	0	0	0	0	5
Mouhoun	7	0	0	0	0	0	0	7
Kénédougou	32	36	1	6	8	0	9	2
Houet	23	10	4	10	4	1	0	11
Léraba	8	0	0	1	0	0	0	61
Comoé	15	0	0	2	1	1	0	2
Poni	6	0	0	0	0	0	0	7
Noumbiel	2	0	0	0	0	0	0	47
Bougouriba	5	0	0	0	2	0	0	1
Ioba	3	0	1	0	0	0	0	9
Sissili	9	0	2	7	1	0	0	5
Nahouri	12	41	4	3	10	12	2	6
Kadiogo	4	2	1	1	0	0	0	44
Boulgou	5	7	0	5	1	1	0	0
Kouritenga	0	2	0	1	1	0	0	1
Koulpélogo	1	0	0	0	0	0	0	21
Gourma	5	1	0	2	2	0	1	8

### 2.2 Nitrocellulose membrane ELISA for virus diagnosis

Fresh leaf samples from stem cuttings growing in the insect‐proof greenhouse were subjected to a nitrocellulose membrane ELISA (NCM‐ELISA) to test for presence of viruses. The NCM‐ELISA test kit with polyclonal antibodies was used according to the manufacturer's instructions and the protocol by Lizarraga and Fernandez‐Northcote (1989). The kit was supplied by the International Potato Center (CIP; Lima, Peru), and can detect the following 10 viruses: SPFMV, SPCSV, sweet potato mild mottle virus, sweet potato latent virus, sweet potato chlorotic flecks virus, sweet potato mild speckling virus, sweet potato C6 virus, sweet potato collusive virus, sweet potato virus G (SPVG), and cucumber mosaic virus. The membranes were prespotted with negative and positive controls of each virus; viral presence was indicated by a colour shift in the membrane of virus‐positive samples.

### 2.3 Reverse transcription PCR and PCR for amplification of *CP* genes

#### 2.3.1 SPFMV and SPCSV

Total RNA was extracted from leaf samples using a RNeasy Plant Mini Kit (Qiagen) according to the manufacturer's instructions. Reverse transcription (RT) was performed on extracted RNA using MMLV reverse transcriptase (Promega) and random hexamers (Promega) as primers at 42 °C for 1 hr (Prasanth and Hegde, 2008) to produce cDNA.

Thereafter, PCR was carried out for the amplification of the SPFMV *CP* gene. The primers used for this step were CP1A (5ʹ‐GCAGAGGATGTCCTATTGCACACC‐3ʹ) and CP1S (5ʹ‐AGTGGGAAGGCACCATACATAGC‐3ʹ), previously described by Prasanth and Hegde (2008), together with Maximo *Taq* DNA polymerase (GeneON). The PCR was carried out in 50 µl reaction volumes using 2.5 µl cDNA and 0.2 µM each of primers CP1A/CP1S. The conditions were 94 °C for 3 min; then 30 cycles of 94 °C for 30 s, 56.3 °C for 30 s, and 72 °C for 1 min; and a final cycle of 72 °C for 10 min.

Primers CP‐F (5ʹ‐ATGGCTGATAGCACTAAAGTCGA‐3′) and CP‐R (5′‐TCAACAGTGAAGACCTGTTCCAG‐3′) were used to amplify the *CP* gene of SPCSV according the protocol of Qin *et al. *(2014). The PCR was carried out in 50 µl reaction volumes using 3 µl cDNA, and 0.2 µM of primers CP‐F/CP‐R using the LA *Taq* DNA polymerase (TaKaRa). The conditions were 94 °C for 3 min; then 30 cycles of 94 °C for 30 s, 58 °C for 30 s, and 72 °C for 45 s; and a final cycle of 72 °C for 10 min.

#### 2.3.2 SPLCV

The 600 dried young leaves collected from the field were processed for DNA extraction. Leaf samples (30 mg) were ground using the TissueLyser II (Qiagen) and DNA was extracted using the CTAB method (Doyle and Doyle, 1987). Degenerate primers SPG1 5′‐CCCCKGTGCGWRAATCCAT‐3′ and SPG2 5′‐ATCCVAAYWTYCAGGGAGCTAA‐3′ developed by Li *et al. *(2004) were used to amplify the *CP* gene of sweepoviruses that infect sweetpotato. The optimized amplification was performed in 50 µl reaction volumes containing 2 µl of the extracted DNA, 1 µl of each primer (10 µM), 1 µl of 10 mM dNTP mix, 5 µl of 10 × *Taq* DNA polymerase reaction buffer, 0.2 µl (1 U) of Maximo *Taq* DNA polymerase and 39.8 µl of water. The PCR conditions used were as follows: 94 °C for 5 min; then 30 cycles of 90 °C for 40 s, 60 °C for 40 s, and 72 °C for 1 min; and a final cycle of 72 °C for 10 min. To confirm amplification, 10 µl of PCR products were electrophoresed in 1% agarose gel in Tris‐acetate buffer (40 mM Tris‐acetate, 1 mM EDTA, pH 8.0), stained with ethidium bromide and viewed under UV transillumination.

### 2.4 Sequencing and sequence analysis

PCR products from RT‐PCR and PCR were sequenced by the Sanger method by Genewiz Company. Contigs obtained were cleaned and assembled de novo using Geneious v. 8.1.7 (Biomatters Ltd). All the sequences were subjected to the BLAST search tools in NCBI using Geneious and subsequently to pairwise sequence comparison (Altschup *et al.*, 1990; Bao *et al.*, 2014). The homologous sequences were retrieved for phylogenetic analysis. Consensus sequences were MAFFT‐aligned using T‐coffee v. 11.00.8 tools (Chang *et al.*, 2014).

Using ClustalW in MEGA v. 7.0.14, the sequences were aligned with sequences from other parts of the world retrieved from GenBank (Kumar *et al.*, 2016). Evolutionary history was inferred using maximum likelihood with the Tamura–Nei model (Kumar *et al.*, 2016). Among a number of models, the Tamura–Nei model provided the best nucleotide substitution fit for our data. Phylogenetic reconstruction was performed with bootstrap support values of 1,000. The trees were visualized and edited using FigTree v. 1.4.3.

### 2.5 Virus transmission by grafting

Sweetpotato samples to be used in grafting to *I*. *setosa* were selected on the basis of the NCM‐ELISA, PCR, and RT‐PCR results. For each of the three virus species detected, scions were taken from 10 sweetpotato plants known to be infected with a single virus. Grafts were also taken from 10 samples each from plants coinfected with SPFMV + SPCSV, SPFMV + SPLCV, SPCSV + SPLCV, or SPFMV + SPCSV + SPLCV. Additionally, we selected five plants grown from symptomless samples and five plants grown from samples with symptoms that gave negative results in all molecular tests. In total, three scions from each of 50 sweetpotato plants were side‐grafted to a healthy *I*. *setosa* plant. All samples grafted were grown in an air‐conditioned, insect‐proof glasshouse maintained at 25–27 °C. The symptoms were recorded weekly for 60 days.

The *I*. *setosa* leaf samples were harvested 25, 45, and 60 days after grafting. All plants were subjected to the same serological and molecular tests described previously for the sweetpotato samples.

## 3 RESULTS

### 3.1 Symptoms observed in fields

**Figure 2:**
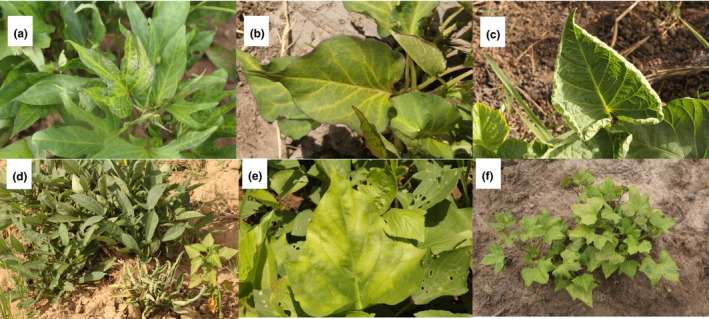
Leaf symptoms on sweetpotato plants affected by viruses. (a, b) Mild mosaic and vein‐clearing. (c) Leaf curling upward at the margin with vein‐clearing. (d) Symptomless plant (extreme left) with severe stunting of growth and leaf curling (extreme right). (e) Chlorotic spotting in systemically infected leaves. (f) Leaf narrowing, stunting of growth, mosaic and mottling symptoms [Colour figure can be viewed at wileyonlinelibrary.com]

The most frequently observed viral symptoms on field plants were stunting, leaf curling, vein‐clearing, and leaf distortion. Of these, the most severe were stunting and leaf curling. Chlorotic spots and purpling were also observed on older leaves. Leaf symptoms among the collected samples were generally mild regardless of geographical location and sweetpotato cultivar. However, certain symptoms were more pronounced in some field plants, showing vein‐clearing (Figure 2a,b), chlorotic spotting (Figure 2e), distortion, leaf curling, and stunting (Figure 2c,d,f) of leaves.

### 3.2 Detection of viruses

The NCM‐ELISA revealed the presence of only two virus species: SPFMV and SPCSV. Of the 600 sweetpotato field samples tested, 45% tested positive for viruses: 34% positive for SPFMV and 11% positive for SPCSV (6% of all plants tested were coinfected with both viruses). About half (55%) of the plants tested negative for all 10 of the virus species that the NCM‐ELISA could detect. Approximately 4% of the 113 symptomless plants were SPFMV‐positive. However, 22% of the 487 plants with symptoms that tested negative for all 10 antisera had leaf curling and leaf distortion symptoms (Figure 2c,d). Using RT‐PCR, we found that 48% gave negative results for the two viruses identified through NCM‐ELISA, 38% were SPFMV‐positive, 14% were SPCSV‐positive (Figure 1b, Table 1), and 7% showed SPFMV + SPCSV coinfection (Figure 1c, Table 1).

Next, PCR was performed on all 600 extracted DNA samples to check for presence of SPLCV; 28% of the sweetpotato plants were positive for SPLCV. Using SPG1 and SPG2 primers to test for SPLCV, 72% of plants were virus‐negative.

The main virus‐detection results from the 600 plants processed are reported in Table 1. Single infections were as follows: 24% plants were singly infected with SPFMV, 18% with SPLCV, and 2% with SPCSV (Table 1). Taking into account all sweetpotato samples tested, we found the following instances of coinfections: 7% with SPFMV + SPCSV, 5% with SPFMV + SPLCV, 3% with SPCSV + SPLCV, and 2% with SPFMV + SPCSV + SPLCV (Table 1). Both the PCR and RT‐PCR results showed that 40% of the samples were virus‐negative.

Plants collected from all areas surveyed showed SPFMV symptoms, mild mosaic, and vein‐clearing (Figure 1b). The plant samples with the most severe symptoms (leaf distortion and stunting) were those coinfected by SPFMV and SPCSV (Figure 1d). SPFMV was the most prevalent virus in all surveyed areas (Figure 1b). In contrast, coinfections of SPLCV + SPCSV (Figure 1d) were found only in areas where sweetpotato production is high.

### 3.3 ****CP**** **gene analysis**

Using de novo assembly in Geneious, 15 partial sequences were obtained from RT‐PCR and PCR products. From these partial sequences, 13 *CP* gene sequences were obtained for SPFMV, one for SPCSV and one for SPLCV (Table 2).

**Table 2:** Isolates and strains of sweet potato feathery mottle virus (SPFMV), sweet potato chlorotic stunt virus (SPCSV), and sweet potato leaf curl virus (SPLCV) from Burkina Faso detected or used for phylogenetic analysis in this study

Virus	Isolate	Strain	Accession no.
SPFMV	BFA1	B (RC)	LT977076
	BFA1b	B (RC)	LT977262
	BFA3	B (RC)	LT977176
	BFA3b	B (RC)	LT977180
	BFA3c	B (RC)	LT977174
	BFA3d	B (RC)	LT977172
	BFA3e	B (RC)	LT977182
	BFA79	A‐II (O)	LT977186
	BFA148	A‐II (O)	LT977184
	BFA169	A‐II (O)	LT977170
	BFA173	B (RC)	LT977178
	BFA412	B (RC)	LT977264
	BFA465	B (RC)	LT977266
SPCSV	BFA1279	WA	LT993430
Sweepoviruses	BFA271	SPLCV‐US	LS991866

Note

SPFMV phylogroups are B (RC), A‐II (O), A‐I (EA); SPCSV strains are West Africa (WA) and East Africa (EA); and sweepoviruses are sweet potato mosaic virus (SPMV), sweet potato leaf curl Canary virus (SPLCCV), sweet potato leaf curl South Carolina virus (SPLCSCV), sweet potato leaf curl Georgia virus (SPLCGV), sweet potato leaf curl Henan virus (SPLCHnV), sweet potato leaf curl Sao Paulo virus (SPLCSPV), sweet potato leaf curl Sichuan virus (SPLCSiV), sweet potato leaf curl South Carolina virus (SPLCSCV), sweet potato leaf curl Uganda virus (SPLCUV), sweet potato leaf curl virus–China (SPLCV‐CN), sweet potato leaf curl virus–Brazil (SPLCV‐BR), sweet potato leaf curl virus–Fujian (SPLCV‐Fu), sweet potato leaf curl virus–Italy (SPLCV‐IT), sweet potato leaf curl virus–Japan (SPLCV‐JP), sweet potato leaf curl virus–Paraiba (SPLCV‐PB), sweet potato leaf curl virus–Pernambuco (SPLCV‐PE), sweet potato leaf curl virus–Puerto Rico (SPLCV‐PR), sweet potato leaf curl virus–Rondonia (SPLCV‐RO), sweet potato leaf curl virus–Sao Paulo (SPLCV‐SP), sweet potato leaf curl virus–South Carolina (SPLCV‐SC), sweet potato leaf curl virus–United States (SPLCV‐US), sweet potato leaf curl virus–Spain (SPLCV‐ES), and sweet potato virus C (SPVC).

The SPFMV amplicons, approximately 945 nucleotides in length and corresponding to the CP‐encoding region, were successfully amplified and sequenced from 13 selected samples using the primer pair CP1A and CP1S. The *CP* sequences from the 13 isolates obtained from plants in Burkina Faso shared more than 99% nucleotide identity with one another. The BLASTn searches of sequences from other studies resulted in three SPFMV groupings, highlighting the identity of Burkina Faso SPFMV sequences with those SPFMV sequences from other geographical locations. Eight Burkina Faso *CP* sequences (LT977172, LT977174, LT977176, LT977180, LT977262, LT927263, LT977264, and LT977265) showed 98% nucleotide identity with SPFMV isolates from Egypt and Korea (GenBank accession numbers AJ515379 and AF015540, respectively). The second group showed Burkina Faso *CP* sequences (LT977178 and LT977266) with 94% nucleotide identity with SPFMV isolates from East Timor and the USA (MF572055 and S43450, respectively). Burkina Faso *CP* sequences LT977170 and LT977184 showed 94% nucleotide identity with SPFMV isolates from Kenya and Peru (AY459593 and EU021065, respectively). In the last group, the *CP* sequence LT977186 showed 96% nucleotide identity with SPFMV isolates from East Timor and Japan (MF572056 and AB465608, respectively). The corresponding translated CP protein sequences from all 13 Burkina Faso sequences contained 315 amino acids with the DAG tripeptide motif at the N‐terminus (amino acid positions 9–11).

A SPCSV amplicon of approximately 774 nucleotides in length and corresponding to the CP‐encoding region was amplified using primers CP‐F and CP‐R and sequenced. The BLASTn search revealed that Burkina Faso sequence LT993430 showed 98% nucleotide identity with two SPCSV isolates from Spain (KU511274 and FJ807785).

A SPLCV amplicon of approximately 792 nucleotides in length, corresponding to the CP‐encoding region, was amplified from selected samples using primers SPG1 and SPG2 (Li *et al.*, 2004) and successfully sequenced. The BLASTn search revealed that sequence LS991866 had 98% nucleotide identity with SPLCV‐US isolate FJ176701 from China.

**Figure 3:**
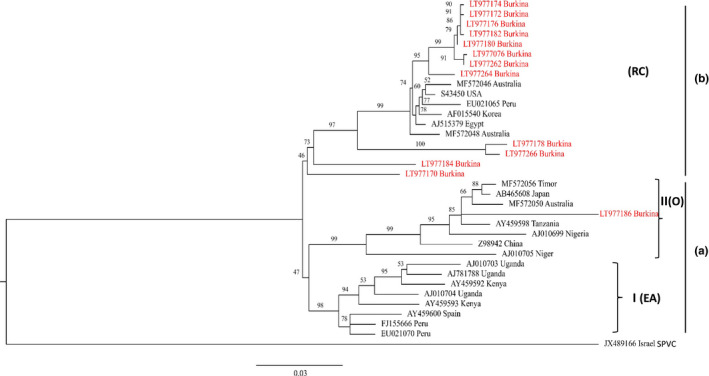
Maximum‐likelihood phylogenetic trees obtained from alignment of nucleotide sequences of coat protein (*CP*) genes from sweet potato feathery mottle virus (SPFMV). The comparisons made were between phylogroup C (three) and *CP* gene (nine) sequences obtained from GenBank, strain O, one new *CP* gene sequence obtained plus the five *CP* gene sequences from GenBank, and phylogroup RC, 10 new *CP* gene sequences obtained plus four *CP* genes sequences from GenBank. The tree was created in MEGA v. 7.0.14 using ClustalW with 1,000 replicates. Bootstrap values are percentages with values shown at the nodes. The tree was rooted with sweet potato virus C (JX489166). Sequence labels: from this study (red), from GenBank (black) [Colour figure can be viewed at wileyonlinelibrary.com]

The *CP* genes from the 13 SPFMV isolates from Burkina Faso were compared with 18 SPFMV isolates from other geographical locations in the phylogenetic analysis, revealing three major phylogroups: A‐I, A‐II, and B (Figure 3). In phylogroup B, sequences of isolates from Burkina Faso (LT977176, LT977172, LT977182, LT977262, LT977180, LT977076, LT977174, LT977264, LT977178, and LT977266) clustered with sequences AJ515379 (Egypt), EU021065 (Peru), S43450 (USA), and AF015540 (Korea). Phylogroup A‐II clustered Burkina Faso isolate sequences LT977186, LT977184, and LT977170 with sequences AY459598 (Tanzania), AJ010699 (Nigeria), AJ010705 (Niger), AB465608 (Japan), and Z98942 (China).

**Figure 4:**
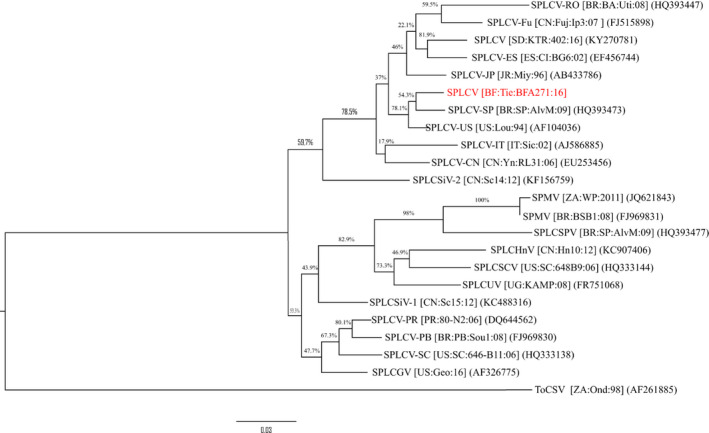
Maximum‐likelihood phylogenetic trees obtained from alignment of nucleotide sequences of coat protein (*CP*) genes from sweet potato leaf curl virus (SPLCV). The comparisons made were between 22 *CP* gene sequences obtained from GenBank and one new *CP* gene sequence obtained in this study. The tree was created in MEGA v. 7.0.14 using ClustalW with 1,000 replicates. Bootstrap values are percentages with values shown at the nodes. The tree was rooted with tomato curly stunt virus (ToCSV) (AF261885). Sequence labels: from this study (red), from GenBank (black) [Colour figure can be viewed at wileyonlinelibrary.com]

The *CP* gene from the partially sequenced SPLCV isolate from Burkina Faso was compared with 24 *CP* genes in SPLCV isolates from around the world. Our phylogenetic analysis clustered the Burkina Faso isolate BFA271 with SPLCV‐US sequences AF104036 (USA) and HQ393473 (Brazil) (Figure 4).

**Figure 5:**
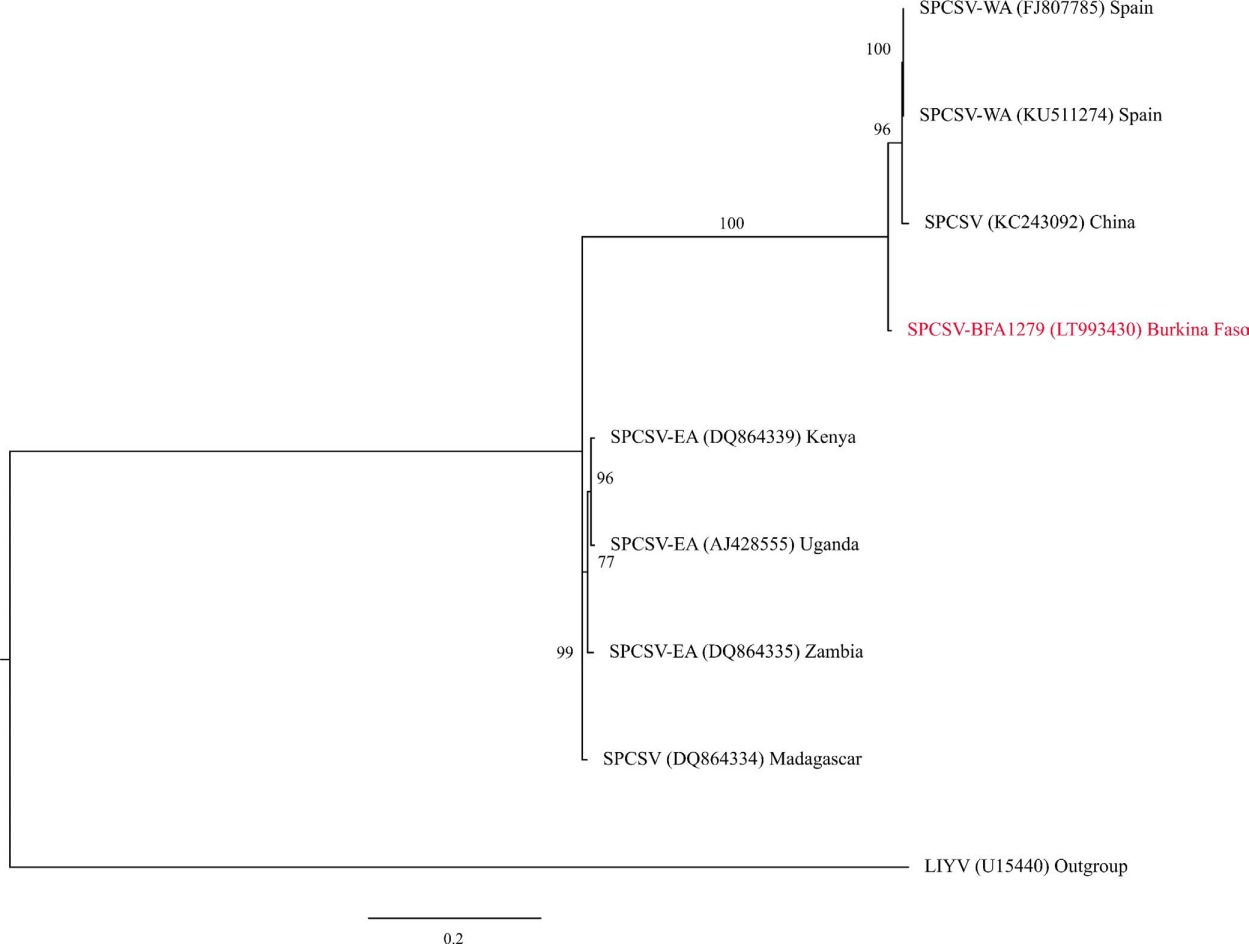
Maximum‐likelihood phylogenetic trees obtained from alignment of nucleotide sequences of coat protein (*CP*) genes from sweet potato chlorotic stunt virus (SPCSV). The comparisons made were between 15 *CP* gene sequences obtained from GenBank and one new *CP* gene sequence obtained in this study. The tree was created in MEGA v. 7.0.14 using ClustalW with 1,000 replicates. Bootstrap values are percentages with values shown at the nodes. The tree was rooted with lettuce infectious yellows virus (LIYV) (U15440). Sequence labels: from this study (red), from GenBank (black) [Colour figure can be viewed at wileyonlinelibrary.com]

The *CP* gene from the partially sequenced SPCSV isolate from Burkina Faso was compared with eight *CP* genes in SPCSV isolates from around the world. Our phylogenetic analysis showed that isolate BFA1279 (sequence LT993430) from Burkina Faso belonged to strain WA together with Spanish sequences KU511274 and FJ807785 (Figure 5).

### 3.4 **Grafting on** ****I. setosa****

The only samples selected for grafting to *I*. *setosa* were those that showed single as well as coinfections as detected through NCM‐ELISA, RT‐PCR, and PCR.

**Figure 6:**
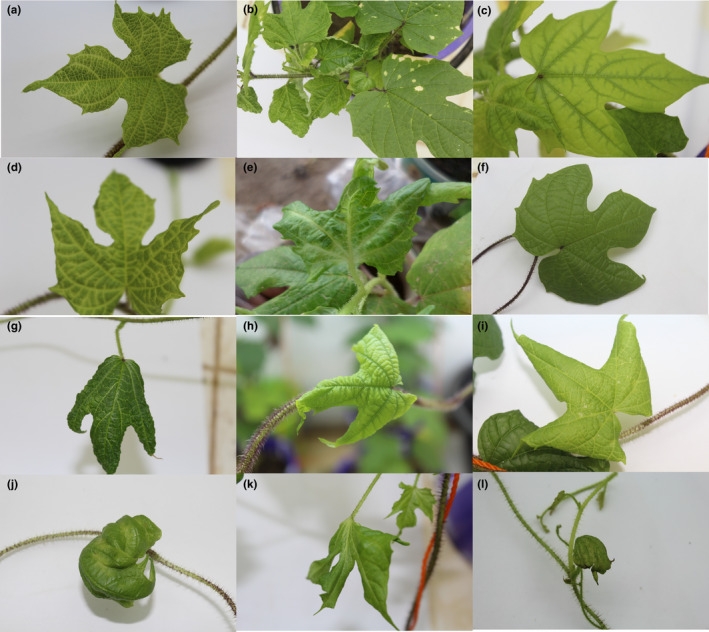
Virus‐associated symptoms in *Ipomoea setosa* plants infected by grafting with *I*. *batatas*. (a) Vein‐clearing like that seen in SPFMV infection; (b) upward leaf curling due to SPLVC; (c) leaf chlorosis due to SPCSV; (d, e) leaf curling, interveinal chlorosis, and blistering due to SPFMV + SPCSV + SPLCV coinfection; (f) symptomless leaf; (g) downward leaf curling and vein‐clearing due to SPFMV + SPLCV coinfection; (h, i) downward leaf curling, chlorosis, and light vein‐clearing due to SPCSV + SPLCV; (j–l) chlorosis, vein‐clearing, severe leaf distortion, blistering, and stunting due to SPFMV + SPCSV (sweet potato virus disease) [Colour figure can be viewed at wileyonlinelibrary.com]

Grafting allowed us to successfully transmit all three viruses identified by molecular analysis to *I*. *setosa* (Table 3) and evaluate plants for viral symptoms (Figure 6). Of the five symptomless sweetpotato samples that were virus‐negative according to the PCR and NCM‐ELISA results, two samples—BFA383 from Di and BFA340 from Bagré—were positive for SPFMV on the grafted *I*. *setosa* plants (according to RT‐PCR).

**Table 3:** Sweetpotato (*Ipomoea batatas*) samples from Burkina Faso were indexed by grafting onto healthy *I. setosa* plants; symptoms were recorded, and viral presence detected using nitrocellulose membrane (NCM)‐ELISA, reverse transcription (RT)‐PCR, and PCR

Locality	Sweetpotato sample	Symptoms		NCM‐ELISA		PCR/RT‐PCR		
		*I. batatas*	*I. setosa*	SPFMV	SPCSV	SPFMV	SPCSV	SPLCV
Bama	BFA44^a^	A	A	−	−	−	−	−
Di	BFA383^a^	A	A	−	−	+	−	−
Douna	BFA1055^a^	A	A	−	−	−	−	−
Bagré	BFA340^a^	A	I	−	−	+	‐	‐
Matourkou	BFA1486^a^	A	Vc	−	−	−	−	−
Bama	BFA49^b^	Cp	A	−	−	−	−	−
Sikorola	BFA96^b^	Vc	Vc, Vn	+	−	+	+	−
Sokouraba	BFA160^b^	C	A	−	−	−	−	−
Tiébélé	BFA199^b^	Ld, Vc, Lc	Ld, B	+	+	+	+	−
Yoro	BFA428^b^	Ld, Vc, Lc	Vn, Np, C	−	−	−	−	−
Mahon	BFA173^c^	Vc, Lc	Vc, Lc	**+**	**+**	**+**	**+**	**+**
Sokouraba	BFA169^c^	Vc, C	Vc, Ld, Vn	**+**	**+**	**+**	**+**	**+**
Samogohiri	BFA148^c^	Vc, Cp	I, Ld, C	**+**	−	**+**	**+**	**+**
Bagré	BFA79^d^	Vc, Ld, B	Np, Cp, Ld	**+**	**+**	**+**	**+**	**+**
Bama	BFA40^d^	Vc	Vc, Ld	**+**	−	**+**	−	**+**
Yoro	BFA412^d^	Vc, C, P	C, Ld	**+**	−	**+**	**+**	−
Bamako	BFA1208^e^	Lc, Cp	Lc, S	+	−	+	−	+
Di	BFA384^e^	Vc, Ld,	Vc, Lc	+	−	+	+	+
Bama	BFA69^f^	C	C, Ld	−	−	−	−	+
Guénon	BFA307^f^	Lc, Cp, I	Lc	−	−	−	−	+
Banfora	BFA1092^g^	I, Vc	Vc, I	+	−	+	−	−
Batié	BFA1163^g^	Vc	I, Vn	+	−	+	−	−
Boura	BFA1284^g^	S	Ld, S	+	−	+	**+**	−
Di	BFA381^g^	Vc, Cp	C, Np	−	−	+	−	−
Farakoba	BFA1473^g^	Vc, Ld	I, C	+	+	+	+	−
Sikorola	BFA87^g^	Vc, Np	Vc	**+**	−	**+**	−	−
Kalwatenga	BFA489^g^	C	A	‐	−	+	−	−
Ouargaye	BFA1388^g^	Lc	A	+	−	+	−	−
Sanké	BFA523^g^	Vc	VC, B	+	−	+	−	−
Sao	BFA1308^g^	C	Vc, B	+	−	+	−	−
Bama	BFA50^h^	Vc, Cp	Lc, Cp	−	−	−	+	−
Bama	BFA62^h^	Vc	Lc	−	+	−	+	−
Bonsrima	BFA321^h^	Cp	A	−	−	−	+	−
Boura	BFA1282^h^	S	Cp, Ld	+	+	+	+	−
Boura	BFA1286^h^	Cp	Vn	−	+	−	+	−
Dano	BFA1298^h^	C	C	−	+	−	+	−
Guénon	BFA311^h^	Cp	Vn, Cp	−	−	−	+	−
Sourou	BFA917^h^	C	Cp, Ld	−	−	−	+	−
Tiébélé	BFA289^h^	Cp	A	−	−	−	−	−
Tiébélé	BFA306^h^	C, Lc	Lc	−	−	−	+	−
Bagré	BFA338^i^	C	Lc, Cp	−	−	−	−	+
Bama	BFA55^i^	Lc, Cp, Ld	C, Np, Ld	−	−	−	−	**+**
Bonsrima	BFA322^i^	C, Lc	S	−	−	+	−	+
Di	BFA388^i^	Cp	S	−	−	−	−	+
Kotoura	BFA774^i^	C, Lc	Lc, Cp, Np	−	−	−	+	+
Louanga	BFA366^i^	Lc	Lc	−	−	−	−	+
Mahon	BFA174^i^	C, Lc	A	−	−	−	−	+
Nakalbo	BFA372^i^	Lc	S	−	−	+	+	+
Pampangou	BFA496^i^	Cp	C	−	−	−	−	+
Tiébélé	BFA193^i^	Lc	C	−	−	−	−	+

Abbreviations: A, symptomless; B, blistering; C, chlorosis; Cp, chlorotic points; I, interveinal chlorosis; Lc, leaf curling; Ld, leaf distortion; Np, necrotic points; S, stunting; Vc, vein‐clearing; Vn, vein necrosis.

^a^ Sweetpotato symptomless, NCM‐ELISA negative.

^b^ Symptoms on both *I. batatas* and on grafted *I. setosa.*

^c^ Coinfection with SPFMV + SPCSV + SPLCV.

^d^ Coinfection with SPFMV + SPCSV.

^e^ Coinfection with SPFMV + SPLCV.

^f^ Coinfection with SPCSV + SPLCV.

^g^ SPFMV single infection confirmed by RT‐PCR.

^h^ SPCSV single infection confirmed by RT‐PCR.

^i^ SPLCV single infection confirmed by PCR.

Samples BFA199 (from Tiébélé) and BFA496 (from Sikorola‐Dierikandougou), with symptoms, tested positive for SPFMV and SPCSV based on RT‐PCR and NCM‐ELISA, whereas both samples tested negative for SPLCV by PCR. Only one plant sample (BFA428 from Yoro) showed leaf distortion and vein‐clearing symptoms (Table 3) after grafting but was virus‐negative according to all three diagnostic tests. Eight of the 10 sweetpotato samples tested positive for single SPFMV infection in our molecular tests; this result was supported by the grafting results on *I*. *setosa,* which exhibited typical SPFMV vein‐clearing symptoms (Figure 6a). The other two samples (BFA1284 from Boura and BFA1473 from Matourkou) showed SPFMV + SPCSV coinfection.

For those sweetpotato samples initially found by PCR and RT‐PCR to be singly infected by SPCSV (Figure 6c), the grafting results confirmed SPCSV infection in 9 of 10 samples. Sample BFA1282 (from Boura) showed coinfection of SPFMV + SPCSV (Figure 6k). No other virus was found in sample BFA289 (from Tiébélé) after grafting onto *I*. *setosa*.

In the cases of those sweetpotato samples determined through molecular analysis to be infected singly by SPLCV, the diagnosis was confirmed by the *I*. *setosa* grafting results that showed SPLCV symptoms (Figure 6b). Mixed infection was found as follows: SPFMV + SPCSV + SPLCV was detected in the plant grafted with BFA372 (Nakalbo; Figure 6d,e), SPFMV + SPLCV was detected in the plant grafted with BFA322 (Bonsrima; Figure 6g), and SPCSV + SPLCV was detected in the plant grafted with BFA774 (Kotoura; Figure 6h,i).

Twenty‐one days after grafting scions on *I*. *setosa*, viral symptoms were visible on 5 out of 150 grafted plants. After 60 days, symptoms varied from moderate to severe. The major symptoms were vein‐clearing, leaf curling, interveinal chlorosis, and chlorosis. The most severe symptoms were in cases of coinfection, typically involving SPFMV + SPCSV (Figure 6j–l), SPFMV + SPLCV, and SPCSV + SPLCV. Surprisingly, coinfections involving all three viruses (SPFMV + SPCSV + SPLCV) only produced moderate or mildly severe symptoms (Figure 6d,e). These symptoms included leaf distortion, vein‐clearing, chlorotic spots, and necrotic spots. After 60 days, three of the five plants grafted with symptomless sweetpotato scions were still symptomless, whereas the other two plants exhibited vein‐clearing and interveinal chlorosis symptoms (Table 3).

## 4 DISCUSSION

Despite studies on virus accumulation in sweetpotato and on the presence of sweet potato viruses in East Africa, the incidence of viruses is poorly documented in many regions and countries of Africa. For our work, we conducted a baseline survey to determine the status of viral diseases in sweetpotato across Burkina Faso. Extensive field surveys (2015–2017) were carried out to assess the prevalence and distribution of viral diseases on sweetpotato plants in the major sweetpotato‐growing areas of Burkina Faso.

Some samples that tested negative using NCM‐ELISA were found to be positive for SPFMV and SPCSV when using RT‐PCR, thus highlighting the importance of using multiple assays for detecting viruses. Because of the known synergy between SPCSV and some DNA viruses (Cuellar *et al.*, 2015), we also checked for the presence of DNA viruses belonging to the genus *Begomovirus* using molecular tools.

To determine which strains of the detected viruses are present in Burkina Faso, we sequenced PCR products for SPFMV, SPCSV, and SPLCV. These results demonstrated that SPFMV A‐II and B phylogroups, SPCSV‐WA, and SPLCV‐US were all present in sweetpotato fields in Burkina Faso. Samples BFA340 from Bagré and BFA383 from Di were negative for viruses in all our diagnostic tests; however, after grafting to *I*. *setosa*, these two plants showed positive results for at least one of SPFMV, SPCSV, and SPLCV. Similar results were obtained by Abad and Moyer (1992) and Valverde *et al* *. *(2007) who proposed that the phenolics and latex found in some sweetpotato cultivars might compromise nucleic acid extraction.

The most widespread virus on sweetpotato crops around the world is SPFMV (Ateka *et al.*, 2004; Njeru *et al.*, 2008; Maina *et al.*, 2018), and it was also the most prevalent virus detected (24%) in our Burkina Faso samples. Leaf vein‐clearing is not specific to SPFMV (Moyer and Salazar, 1989) but can indicate the presence of other viral infections. Because mosaic and streak‐like symptoms were also prevalent in our sweetpotato samples from fields, it is likely that other viruses in the family *Potyviridae* were also present (Moyer and Salazar, 1989; Brunt *et al.*, 1996).

The second most prevalent virus that we detected was SPLCV (18%). As a single infection, SPCSV was also present among samples collected but was not very common in the areas surveyed. However, we did observe instances of SPFMV + SPCSV coinfection, a cause of SPVD. This viral disease has been reported as having the greatest impact on sweetpotato yield worldwide (Valverde *et al.*, 2007; Rey *et al.*, 2012; Loebenstein, 2015). The low prevalence of SPCSV (2%) in our survey areas might mask its true impact if infections are symptomless and thus may also represent a potential threat to sweetpotato production in Burkina Faso. Molecular studies have shown that coinfection of SPCSV enhances SPFMV RNA viral titres by at least 600‐fold, whereas SPCSV titres remain equal or are reduced compared to single infection (Karyeija *et al.*, 2000; Mukasa *et al.*, 2006; Untiveros *et al.*, 2008). The presence of purple pigmentation, strapping, stunting, and puckering symptoms often result from SPFMV + SPCSV coinfection (Ndunguru and Kapinga, 2007). Some of their findings were applicable to our work: our samples from stunted sweetpotato plants were found to be coinfected by SPFMV and SPCSV in Kenedougou, Houet, Nahouri, and Sissili provinces. Our mixed infections (16%) involving SPFMV + SPCSV + SPLCV, SPFMV + SPCSV, SPFMV + SPLCV, and SPCSV + SPLCV reflect those reported in East Africa (Kenya, Rwanda, Tanzania, and Uganda), the USA, and Korea (Ateka *et al.*, 2004; Clark and Hoy, 2006; Mukasa *et al.*, 2006; Njeru *et al.*, 2008; Cuellar *et al.*, 2015; Kim *et al.*, 2017). Few reports exist on the synergistic interactions between RNA and DNA viruses. However, previous studies, such as Cuellar *et al. *(2015), showed that SPCSV can interact synergistically with sweepoviruses.

Grafting sweetpotato scions onto *I*. *setosa*, and subsequent use of diagnostic tests, confirmed the presence of the three virus species and of coinfections; the *I*. *setosa* also displayed the symptoms associated with each virus (Table 3). Two sweetpotato samples with symptoms gave negative results in the diagnostic tests, but *I*. *setosa* plants grafted with these samples produced clear symptoms of viral infection. The possible interaction of these viruses with SPFMV has been shown in the work of Clark *et al. *(2012) and Mulabisana *et al. *(2018).

The majority of symptoms observed in this study are in accordance with the typical SPFMV symptoms described by Moyer and Salazar (1989) and Gibson *et al.* (1997). Previous research confirmed that SPFMV on its own causes mild or no symptoms in some sweetpotato cultivars (Gibson *et al.*, 1997; Ateka *et al.*, 2004), suggesting that the SPFMV‐positive, symptomless samples in our study may have been singly infected with SPFMV. The most severe symptoms were observed for coinfections, mainly involving SPFMV + SPLCV but sometimes SPCSV + SPLCV. These results corroborate those of previous studies using grafting on *I*. *setosa* (Maina *et al.*, 2018). In addition, our study showed that the accumulation of three or more viruses does not necessarily have a more devastating effect than a coinfection involving only two viruses. Indeed, the symptoms observed in double coinfections (SPFMV + SPCSV) were more severe than those resulting from multiple coinfection with SPFMV + SPCSV + SPLCV, for which foliar symptoms were moderate.

Symptomless infections present a challenge for SPFMV management. Fortunately, this virus can be diagnosed by grafting on *I*. *setosa*, even if viral titre is very low and the virus is undetectable by NCM‐ELISA or RT‐PCR. Our study corroborates the results of Gibson *et al. *(1997). They showed that although about 95% of symptomless plants were virus‐negative when NCM‐ELISA was used, in the remaining 5% the most prevalent virus was SPFMV. Moreover, they found that approximately 15% of the symptomless plants tested induced viral symptoms when grafted on *I*. *setosa* (Moyer and Salazar, 1989; Gibson *et al.*, 1997).

Although symptom scoring, serology, and grafting are useful for the detection of SPFMV, SPCSV, and SPLCV, they are not suitable for virus strain demarcation (Ryu and Choi, 2002; Prasanth and Hegde, 2008; Qin *et al.*, 2014). Previous studies showed that SPFMV phylogroup B and phylogroup A are unrestricted geographically (Kreuze *et al.*, 2000; Untiveros *et al.*, 2008; Tugume *et al.*, 2010). Phylogroup B is also known to infect sweetpotato in Asia (Japan, Korea, and China) and North Africa (Egypt) (Tairo *et al.*, 2005). To avoid geographical and biological confusion, Jones and Kehoe (2016) proposed the current approach in which phylogroup nomenclature is based on letters of the alphabet and Latinized numbers.

Our study shows that all SPFMV isolates from Burkina Faso can be assigned to phylogroups A‐II and B. Phylogroup A‐I was not detected among our samples, even though some sweetpotato cultivars have been introduced from East Africa (Somé *et al.*, 2015). Another finding from our examination of CP amino acid sequences of all isolates from Burkina Faso is the presence of the DAG motif that, according to other research (Shukla *et al.*, 1994; Ateka *et al.*, 2017), is involved in virus transmission by aphids. The presence of the DAG motif in the CP, together with the presence of aphids in the environments sampled, suggests that these insects may play a role in SPFMV transmission on sweetpotato in Burkina Faso. We did not investigate viral transmission by aphids, but suggest that this should be considered in future surveys.

Of the samples in this study, 60% were infected with viruses that lead to SPVD, either as single infections or as coinfections, on cultivated sweetpotato in Burkina Faso. These viruses do not seem to be widespread in the surveyed areas; however, their presence—together with that of other viral diseases—could compromise the health of sweetpotato crops in Burkina Faso. Our work confirms that of Ryu and Choi (2002) on the importance of using multiple methods for virus screening, such as NCM‐ELISA, RT‐PCR, and PCR for the detection of SPFMV, SPCSV, and SPLCV.

In view of our results, we conclude that management approaches for SPVD should continue to include monitoring sweetpotato crops in Burkina Faso and should be performed more widely in West Africa. Furthermore, we advocate the setting up of management plans to address the development of resistant varieties and to disseminate information about good farming practices to better control spread of viral diseases. The next step in this project is to obtain the full genomes of SPFMV, SPLCV, and SPCSV. Then we will investigate the mechanisms of mixed infection and test for evidence of synergy to better understand the interactions between DNA and RNA viruses and between viruses and plants.

## CONFLICT OF INTEREST

The authors declare that they have no conflict of interest.

## Supporting information

Table S1

## Data Availability

The data that support the findings of this study are available from the corresponding author upon reasonable request.
