# Supplementary material for: Characterization of virus species associated with sweetpotato virus diseases in Burkina Faso
Source: Plant Pathol. 2020 Apr 30;69(6):1003–17. doi: 10.1111/ppa.13190 (PMC7386933; doi:10.1111/ppa.13190)
Supplement: Supplementary file 1 — Table S1 [file PPA-69-1003-s001.docx]

**Table S1** Isolates and strains of sweet potato feathery mottle virus (SPFMV), sweet potato chlorotic stunt virus (SPCSV) and sweet potato leaf curl virus (SPLCV) used for phylogenetic analysis in this study.

| **Virus isolate** | **Strain** | **Geographical origin** | **Accession no.** |
| --- | --- | --- | --- |
| **SPFMV:** |  |  |  |
| 54/9S | AI (EA) | Kenya | AY459592 |
| 85/7S | AI (EA) | Kenya | AY459593 |
| Bau | AII (O) | Nigeria | AJ010699 |
| Canar3 | AI (EA) | Spain | AY459600 |
| CH | AII (O) | China | Z98942 |
| Eg9 | B (RC) | Egypt | AJ515379 |
| Fe | AI (EA) | Peru | EU021070 |
| Fio | B (RC) | Peru | EU021065 |
| K1 | B (RC) | Korea | AF015540 |
| Mbl2 | AI (EA) | Uganda | AJ781788 |
| Nam1 | AI (EA) | Uganda | AJ010704 |
| Nam6 | AI (EA) | Uganda | AJ010703 |
| Nig3 | AII (O) | Niger | AJ010705 |
| O | AII (O) | Japan | AB465608 |
| Piu3 | AI (EA) | Peru | FJ155666 |
| RC | B (RC) | USA | S43450 |
| Aus7D | B (RC) | Australia | MF572046 |
| Aus11D | B (RC) | Australia | MF572048 |
| TM66B | AII (O) | Timor | MF572056 |
| Aus13B | AII (O) | Australia | MF572050 |
| TZ4 | B (RC) | Tanzania | AY459598 |
| Outgroup | SPVC |  | JX489166 |
| **SPCSV:** |  |  |  |
| EA2 | EA | Uganda | AJ428555 |
| Kakamega1 | EA | Kenya | DQ864339 |
|  | WA | Israel | EU124491 |
| Can181-9/AM-MB2 | WA | Spain | KU511274 |
| Can181-9 | WA | Spain | FJ807785 |
| Zambia1 | EA | Zambia | DQ864335 |
| Madagascar | EA | Madagascar | DQ864334 |
| 92 | Outgroup | USA | U15440 |
| **Sweepoviruses:** |  |  |  |
| [ES-CI-BG4-02] | SPLCCV | Spain | EF456742 |
| [US-Geo-16] | SPLCGV | USA | AF326775 |
| [CN-Hn10-12] | SPLCHnV | China | KC907406 |
| [BR-SP-AlvM-09] | SPLCSPV | Brazil | HQ393477 |
| [CN-Sc15-12] | SPLCSiV-1 | China | KC488316 |
| [CN-Sc14-12] | SPLCSiV-2 | China | KF156759 |
| [US-SC-648-B9-06] | SPLCSCV | USA | HQ333144 |
| [UG-KAMP-08] | SPLCUV | Uganda | FR751068 |
| [CN-Yn-RL31-06] | SPLCV-CN | China | EU253456 |
| [BR-PA-Bel1-08] | SPLCV-BR | Brazil | FJ969829 |
| [CN-Fuj-Ip3-07] | SPLCV-Fu | China | FJ515898 |
| [IT-Sic-02] | SPLCV-IT | Italy | AJ586885 |
| [JR-Miy-96] | SPLCV-JP | Japan | AB433786 |
| [BR-PB-Sou1-08] | SPLCV-PB | Brazil | FJ969830 |
| [BR-RO-PV-08] | SPLCV-PE | Brazil | HQ393456 |
| [PR-80-N2-06] | SPLCV-PR | Puerto Rico | DQ644562 |
| [BR-BA-Uti-08] | SPLCV-RO | Brazil | HQ393447 |
| [BR-SP-AlvM-09] | SPLCV-SP | Brazil | HQ393473 |
| [US-SC-646-B11-06] | SPLCV-SC | USA | HQ333138 |
| [ES-CI-BG6-02] | SPLCV-ES | Spain | EF456744 |
| [US-Lou-94] | SPLCV-US | USA | AF104036 |
| [BR-BSB1-08] | SPMV | Brazil | FJ969831 |
| [SD: KTR: 402-16] | SPLCV | Sudan | KY270781 |
| [ZA: WP:2011] | SPMV | South Africa | JQ621843 |
| Outgroup | [ZA: ToCSV:04] | South Africa | AF261885 |

SPFMV phylogroups are B (RC), AII (O), AI (EA); SPCSV strains are West Africa (WA) and East Africa (EA); and sweepoviruses are sweet potato mosaic virus (SPMV), sweet potato leaf curl Canary virus (SPLCCV), sweet potato leaf curl South Carolina virus (SPLCSCV), sweet potato leaf curl Georgia virus (SPLCGV), sweet potato leaf curl Henan virus (SPLCHnV), sweet potato leaf curl Sao Paulo virus (SPLCSPV), sweet potato leaf curl Sichuan virus (SPLCSiV), sweet potato leaf curl South Carolina virus (SPLCSCV), sweet potato leaf curl Uganda virus (SPLCUV), sweet potato leaf curl virus - China (SPLCV-CN), sweet potato leaf curl virus - Brazil (SPLCV-BR ), sweet potato leaf curl virus - Fujian (SPLCV-Fu), sweet potato leaf curl virus – Italy (SPLCV-IT), sweet potato leaf curl virus - Japan (SPLCV-JP), sweet potato leaf curl virus - Paraiba (SPLCV-PB), sweet potato leaf curl virus - Pernambuco (SPLCV-PE), sweet potato leaf curl virus - Puerto Rico (SPLCV-PR), sweet potato leaf curl virus - Rondonia (SPLCV-RO), sweet potato leaf curl virus - Sao Paulo (SPLCV-SP), sweet potato leaf curl virus - South Carolina (SPLCV-SC), sweet potato leaf curl virus - United States (SPLCV-US), sweet potato leaf curl virus - Spain (SPLCV-ES), sweet potato virus C (SPVC)
